# From medical imaging data to 3D printed anatomical models

**DOI:** 10.1371/journal.pone.0178540

**Published:** 2017-05-31

**Authors:** Thore M. Bücking, Emma R. Hill, James L. Robertson, Efthymios Maneas, Andrew A. Plumb, Daniil I. Nikitichev

**Affiliations:** 1 Department of Medical Physics and Biomedical Engineering, University College London, London, United Kingdom; 2 Centre for Medical Imaging, University College London, London, United Kingdom; University of Pennsylvania, UNITED STATES

## Abstract

Anatomical models are important training and teaching tools in the clinical environment and are routinely used in medical imaging research. Advances in segmentation algorithms and increased availability of three-dimensional (3D) printers have made it possible to create cost-efficient patient-specific models without expert knowledge. We introduce a general workflow that can be used to convert volumetric medical imaging data (as generated by Computer Tomography (CT)) to 3D printed physical models. This process is broken up into three steps: image segmentation, mesh refinement and 3D printing. To lower the barrier to entry and provide the best options when aiming to 3D print an anatomical model from medical images, we provide an overview of relevant free and open-source image segmentation tools as well as 3D printing technologies. We demonstrate the utility of this streamlined workflow by creating models of ribs, liver, and lung using a Fused Deposition Modelling 3D printer.

## Introduction

Anatomical models have applications in clinical training and surgical planning as well as in medical imaging research. In the clinic, the physical interaction with models facilitates learning anatomy and how different structures interact spatially in the body. Simulation-based training with anatomical models reduces the risks of surgical interventions [1], which are directly linked to patient experience and healthcare costs. For example, improvement of central venous catheter insertions has been achieved by the use of anatomically and ultrasonically accurate teaching phantoms [2]. In addition, the phantoms can be used for pre-operative surgical planning, which has been shown to be beneficial in craniofacial surgery [3] and is being explored in a number of other surgical fields [4, 5]. Lastly, anatomical phantoms can be designed to mimic tissue when imaged with the modality of interest; most commonly ultrasound, Computed Tomography (CT), or Magnetic Resonance Imaging (MRI). Imaging phantoms are also important for the development of novel imaging modalities such as photoacoustics [6], or for validation of image-based biomarkers such as pore size estimation using nuclear magnetic resonance [7], where they provide controlled experimental environments.

Anatomically accurate models can be computer-generated from medical image data. CT and MRI are widely used to image biological features, ranging from whole-body imaging to particular areas of interest such as tumours or specific parts of the brain. Depending on the imaging modality, different features can be observed and different image segmentation algorithms will be appropriate. CT pixel intensities directly correlate to tissue density. The modality thus lends itself well to segmenting structures such as bones (high density) or lungs (low density). MRI offers excellent soft tissue contrast, which, for example, enables differentiation between white and grey matter in the brain [8].

Recent advances in segmentation software have made it increasingly easy to automatically or semi-automatically extract the surface of structures of interest from three-dimensional (3D) medical imaging data. This has made it possible to generate anatomical models using a standard personal computer with little prior anatomical knowledge. At the same time 3D printers, traditionally used in industrial applications, are now available for home use thanks to low-cost desktop alternatives. This technology enables fast creation of 3D models without the need for classical manufacturing expertise.

Accessibility to 3D printers and advanced segmentation algorithms have led to an increase in use of 3D printing in medicine, which has received interest due to a multitude of potential medical applications [4, 9]. Models can be made patient-specific, and rapidly redesigned and prototyped, providing an inexpensive alternative to generic commercially available anatomical models. 3D printing thus found applications in teaching the structure of kidney [5], heart [10], and liver [11]. A number of studies have also investigated the potential of using 3D printing techniques to produce tissue-mimicking phantoms for research and teaching, with example applications in producing models of vessels [2, 12], parts of the skull [13], optic nerves [14], and renal system [15]. The process of going from medical imaging data to 3D printed models has been described for the brain [16,17], the human sinus [18], as well as from a general point of view [19], but challenges remain to make the process widely available to novice users.

In this work, we present a practical guide to creating a broad range of anatomical models from medical imaging data. In the next section, we provide an overview of the general workflow and include a table listing the relevant 3D printing technologies. We have implemented a streamlined processing pipeline on various examples to illustrate the different approaches that can be followed. We have developed 3D printed models of ribs, a liver, and a lung. Ribs and lung were chosen as they have complex structure, while the liver illustrates the potential of segmenting and printing soft-tissue organs which have lower contrast with the surrounding tissue in CT images. Finally, we introduce and discuss the currently freely available segmentation tools, which can be applied to any organ or region of interest.

## Materials and methods

### The general workflow

**Fig 1:**
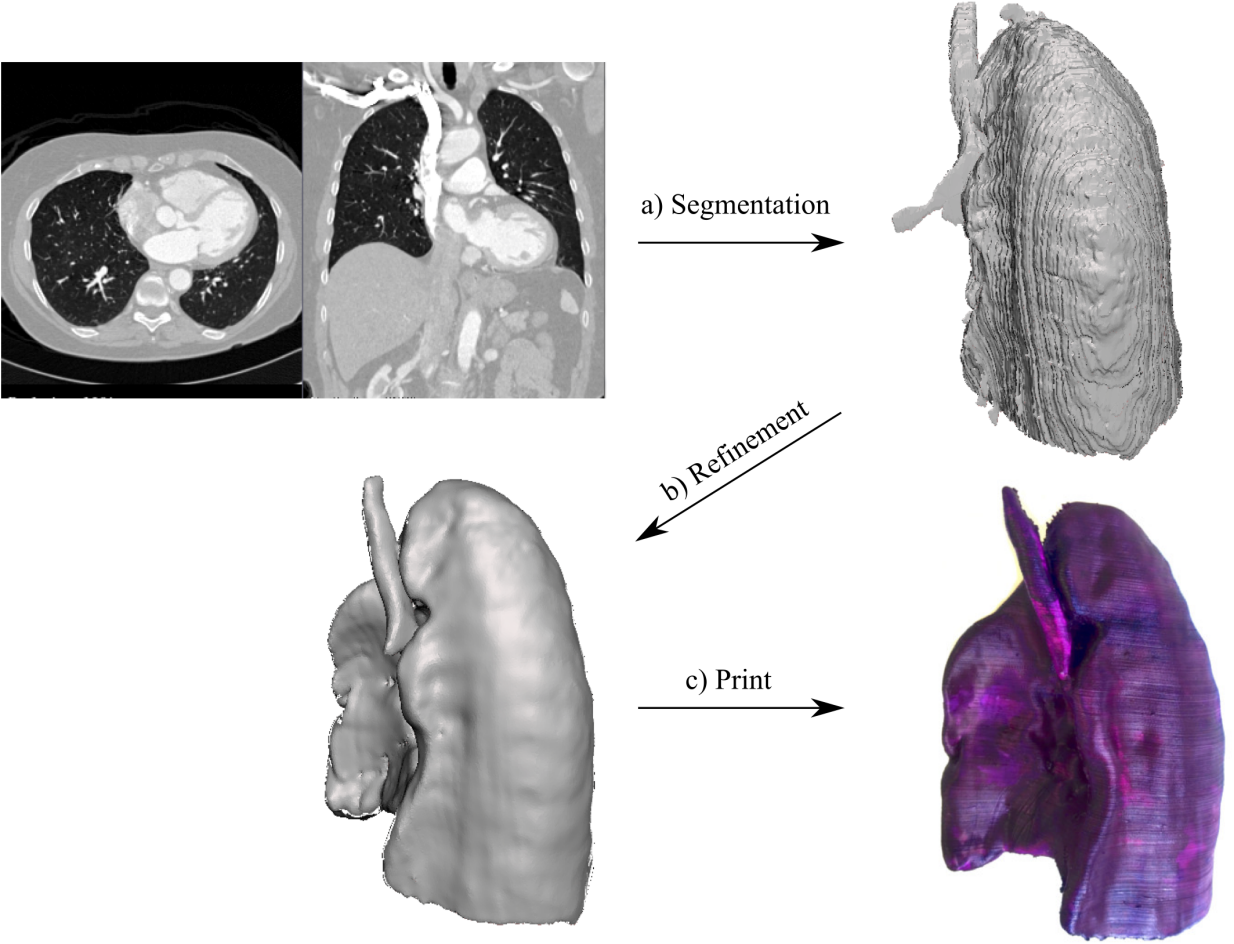
From medical image to 3D print workflow. After the anatomical structure has been segmented (a), the resulting surface needs to be refined (b) to remove image artefacts, after which it can be 3D printed (c).

In this section, we describe the process of going from medical imaging data (CT scan) to a finished 3D printed model from a general point of view. We have implemented a streamlined pipeline on different regions of interest (ribs, liver, and lung), which will be described in the sections below. While the pipeline is illustrated using CT images, it is also applicable to other volumetric medical imaging modalities, like MRI. The workflow is broken down into three steps (Fig 1):

#### Image segmentation

After acquiring a medical image, structures of interest need to be segmented. Image segmentation is the process of partitioning an image into multiple labelled regions locating objects and boundaries in images. It can be used to create patient-specific, highly accurate computer models of organs and tissue. There are a number of image segmentation techniques, which each have advantages and disadvantages, but there is no single segmentation technique which is suitable for all images and applications. Basic segmentation approaches rely on the principle that each tissue type has a characteristic range of pixel intensities. Hence, it is possible to distinguish between tissues and identify boundaries.

There is a wide range of software that is capable of performing image segmentation, ranging from multi-purpose commercial platforms with integrated physics simulations (e.g. Mimics [20], or Simpleware [21]), to open-source tools targeted to specific organs (e.g. FreeSurfer [22] for the brain). In this work, we have used the freeware software packages called Seg3D [23] and 3D Slicer [24], as they are capable of processing a range of medical imaging data. Furthermore, we provide a summary of comparable freeware software available at the time of writing in the discussion.

#### Mesh refinement

Following image segmentation, the 3D model can be further refined into a printable 3D mesh. There are a number of computer-aided design tools that can be used for this purpose and allow almost limitless mesh manipulation and refinement. However, the main reasons for such mesh post-processing of the segmentation are as follows:

Repairing: Errors and discontinuities that sometimes arise in the image segmentation and exporting process need to be repaired before printing.

Smoothing: Staircasing errors resulting from the resolution of the original medical image can be mitigated by smoothing the surface of the mesh model.

Appending: The segmentation will often only be one component of a final model. To convert the model into a useable form, it is often necessary to combine it with other structures or remove unneeded parts from the segmentation.

#### 3D printing

There are many different 3D printing technologies available, each with their own characteristics. Here we provide an overview of the 3D printing methods, which are suitable for the creation of anatomical models and highlight their respective advantages. The relevant 3D printing technologies can be classified into three groups: extrusion printing, photopolymerisation and powder-based printing. The most common example of extrusion printing is known as Fused Deposition Modelling (FDM), which is based on melting and depositing a material via a nozzle, building the desired shape layer by layer. In photopolymerisation, liquid polymers are selectively cured, typically using UV light. Important examples are Stereolithography (SLA) and Digital Light Processing (DLP), which selectively cure a plastic in a bath. Moreover, the photopolymer can be sprayed onto the print in thin layers, where it is subsequently cured. This technique is known as Material Jetting (MJ). Lastly, in powder-based techniques, a powdered material is bound together. This can either be done using a liquid binding agent (Binder Jetting, BJ), or by fusing the particles together using heat (Selective Laser Sintering, SLS). The characteristics of these techniques are summarised in Table 1.

**Table 1:** Overview of the most important 3D printing technologies with medical applications: Extrusion printing, photopolymerisation, and powder binding.

Printing techniques	Advantages	Disadvantages	Examples of medical application
Extrusion printing: Filament Deposition Modelling (FDM)	• Low material costs • Low cost printers available • Simple to use	• Rippled and porous surface • Fragile along z-axis	• Kidney [15] • Liver [11] • Sinus [13,18]
Photopolymerisation: Stereolithography (SLA) & Digital Light Processing (DLP)	• Moderate cost • Good surface finish / high resolution	• Prints are prone to slight distortions • Curing resins need to be handled with care	• Prosthetics [9]
Photopolymerisation: Material Jetting (MJ)	• Very good surface finish / high resolution • Ability to gradually combine different polymers	• High material cost • Curing resins need to be handled with care	• Vessels [12,25] • Spine [26]
Powder Binding: Binder Jetting (BJ)	• Can include colour • Quick • Low material costs • Many materials available	• Printers are expensive • Rough surface finish	
Powder Binding: Selective Laser Sintering (SLS)	• Prints are strong • Many materials available	• Printers are expensive • Rough surface finish	• Brain [16] • Heart [10]

### Preparation of example anatomical models

The models of the ribs and liver were segmented using Seg3D (v.2.2.1) while the lung was segmented using 3D Slicer (v.4.6). We smoothed the models using MeshMixer [27] (v.3.0) and printed all of them using Filament Deposition Modelling (FDM). All processing was done using Windows 10 as operating system.

#### Image segmentation

Seg3D v.2.2.1 was used to generate the rib model from the CT MECANIX dataset (Siemens Sensation 64, 3 mm slice thickness, 0.56 mm by 0.56 mm pixel size, 120 kV peak kilo-voltage, 100 mAs exposure) available on the OSIRIX website [28]. The ribs were segmented using thresholding, manual modification (cropping), a connected-component-filter as well as a fill-holes-filter. The segmentation was exported as a stereolithography (STL) file using the “Export Isosurface” command.

3D Slicer v.4.6 was used to create a model of the liver and the right lung from the CT ARTIFIX dataset (Siemens Sensation 64, 1.5 mm slice thickness, 0.59 mm by 0.59 mm pixel size, 120 kV peak kilo-voltage, 300 mAs exposure) from the OSIRIX website [28]. This was done using the level tracing algorithm as well as manual modification. After segmentation, the “make model” tool was used to export the volume as a STL file.

#### Mesh refinement

The ribs model was refined using Meshmixer to improve its topology. This was done by adjusting the mesh density of the surface and by applying a global smoothing filter that removes step artefacts due to the finite voxel size. Furthermore, we have used FreeCAD (v.0.16) [29] to design a holder for a tissue phantom. As FreeCAD has limitations working with large mesh files, the holder was attached to the ribs model structure in another software package called Blender (v.2.6) [30].

The lung model was also smoothed using MeshMixer. The different segmentation method demanded a local smoothing approach utilising the “RobustSmooth” brush provided by the software. Furthermore, the “Flatten” and “Inflate” brushes were used to remove unphysiological holes in the model.

The smoothing of the liver was also done utilizing both a global smoothing filter as well as the “RobustSmooth” brush tool.

#### 3D printing

We have used an Ultimaker 2 (Ultimaker, Chorley, England) FDM printer to create our models. They were prepared for the printer using the open-source slicing software Cura (v.15.04.6), which is provided for free by Ultimaker. All models were printed with a layer height of 0.12 mm and a shell, bottom, and top thickness of 0.8 mm with a nozzle size of 0.4 mm. The prints were created at 20% infill, except for the rib model, which was printed with 100% infill to be functionally similar to bone when viewed under a medical ultrasound scanner (Siemens Acuson S1000 with a 16 MHz ultrasound probe). The material used for printing was “enhanced Polymax” polylactic acid (PLA) (PolyMax; Polymakr, Changshu, China). To estimate the print accuracy, the dimensions of the models were quantified at different sites *in silico* using Meshmixer and compared to the dimensions of the 3D prints, which were measured using calipers and a micrometer.

## Results and discussion

**Fig 2:**
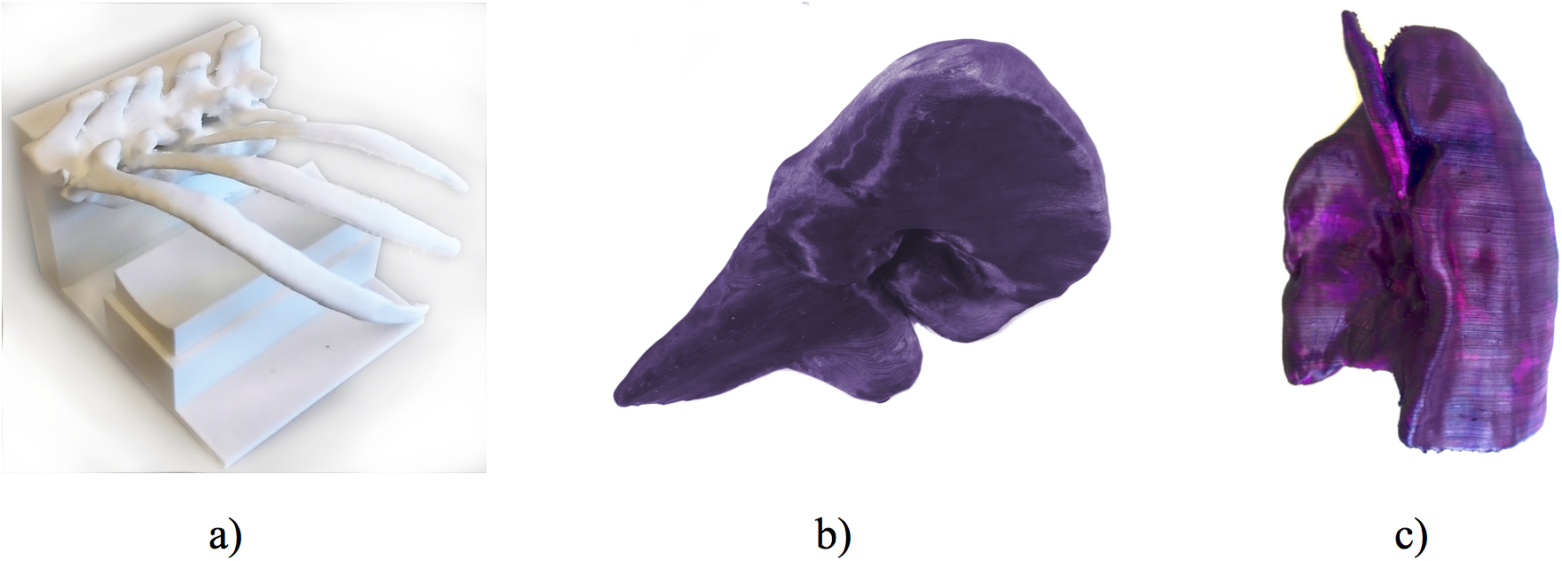
3D printed anatomical models generated from medical imaging data using 3D Slicer and Seg3D. Part of the ribcage (a), the liver (b), and the right lung (c).

The 3D printed models of the ribs, liver and lung can be seen in Fig 2. On the ribs phantom the holder can be seen, which can be used to place tissue mimicking phantoms underneath the ribs in order to perform realistic ultrasound imaging experiments and training. The liver phantom was printed in coloured PLA, while the lung was painted using acrylic colour to be used as a teaching model.

The print duration for lung, ribs, and liver were 43h, 87.5h, and 27.5h respectively. The cost of the PLA for each of the models was approximately £16, £25, and £10 respectively. The print accuracy of the models is summarised in Table 2.

**Table 2:** Quantification of print accuracy based on comparing size of anatomical landmarks between computer model and 3D print.

Phantom	Name of Feature	Measured *in silico* (mm)	Measured on 3D print (mm)	Percentage Error
Ribs	Thickness of superior rib	14.4	14.1	2.1%
	Distance between spinous processes	110.1	110.4	0.3%
	Depth of the spine	75.8	76.2	0.5%
	Length of middle rib	187.2	187.5	0.2%
	**Mean Error**			**0.78%**
Liver	Total height	99.5	99.1	0.4%
	Total width	201.1	198	1.6%
	Total depth	135.1	132.5	1.9%
	**Mean Error**			**1.3%**
Lung	Length of bronchus	75.2	74	1.6%
	Thickness of bronchus	10.8	10.5	2.9%
	AP of base of lung	114.5	116	1.3%
	AP of pericardium	57.5	60	4.3%
	**Mean Error**			**2.53%**

**Fig 3:**
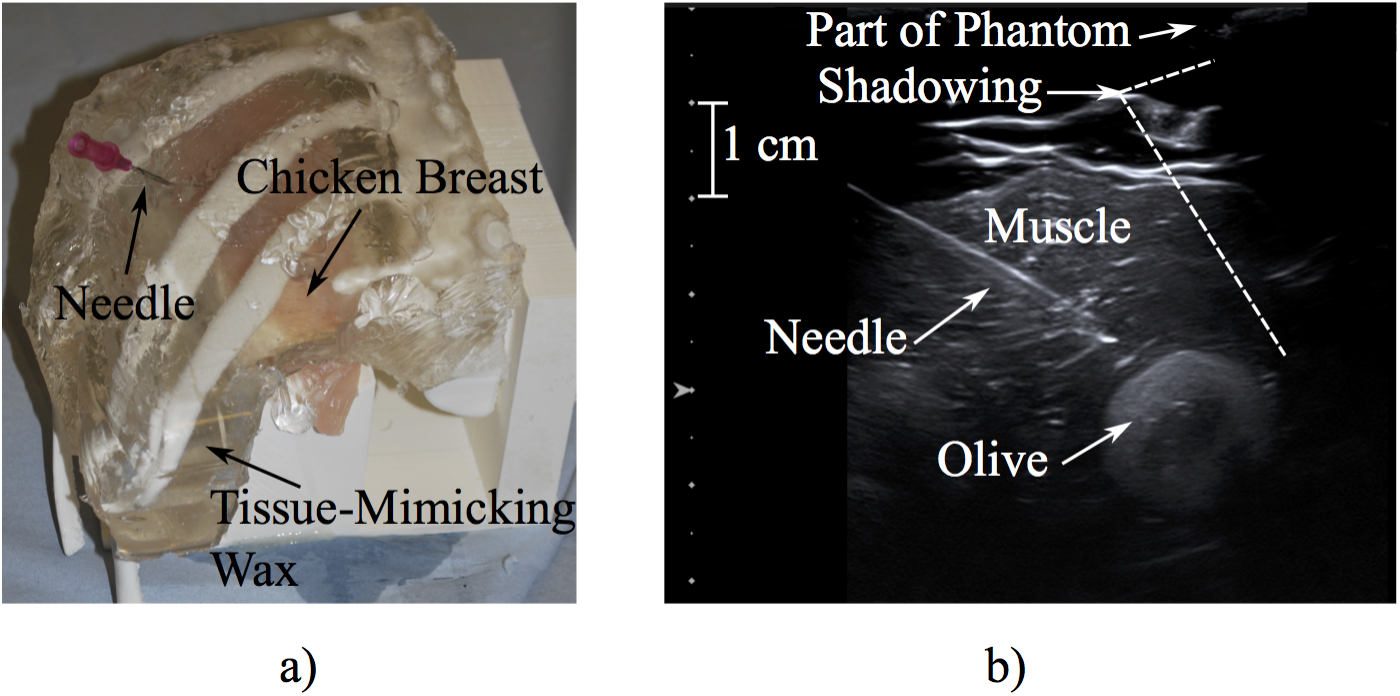
Ribs phantom as a clinical training tool for ultrasound guided kidney biopsy. a) 3D print of the ribs model with a chicken breast and biopsy needle b) Ultrasound scan of the model immersed in water.

The ribs phantom was used as an ultrasound imaging phantom for clinical training. By embedding the ribs into a mineral-oil based material (Mindsets, Waltham Cross, United Kingdom) to simulate surrounding musculature and soft tissues, and combining these with a chicken breast and an 18 gauge puncture needle, it was possible to perform a low-cost mock kidney fine needle aspiration (FNA) procedure (Fig 3) [31]. In Fig 3B, the reflection of part of the ribs phantom can be seen in the top right corner. The artificial ribs are creating a shadowing effect which can also be observed in real ultrasound imaging procedures.

When going from medical imaging data to 3D printed anatomical models, the choice of an appropriate image segmentation algorithm is arguably the most important step. An overview of relevant freeware segmentation tools can be seen in Table 3. We have illustrated the use of two different open-source tools, which offer a multitude of ways to achieve accurate segmentation.

**Table 3:** Overview of freeware software with segmentation tools applicable to any part of the body.

Software name	Segmentation tools	Additional features and comments
Seg3D [23]	• Manual modification • Thresholding • Edge detection (Canny edge filter) • Level Sets • Connected component filter	• Multiple segmentations possible • Intuitive layer based interface • Tools available to edit image and segmentations (e.g. erosion, hole filling, and Boolean combinations of segmentations) • Can compute distance maps
3D Slicer [24]	• Manual modification • Thresholding • Edge detection (Watershed filter) • Fast marching method • Grow cut method • Level tracing method • Range of modified filters for specific tasks (e.g. airway or tumour segmentation)	• Image registration • Changes input data, so copy of data is required • Popular for 3D visualisation
InVesalius [32]	• Manual modification • Thresholding	• Simple interface • Automatic thresholding for bone from CT • Popular for 3D visualisation
ITK-Snap [33]	• Manual modification • Edge detection (active contour methods)	• Simple interface • Multiple segmentations possible
Osirix Lite [34] or Horos [35]	• Manual modification • Edge detection (region growing)	• Macintosh only • For visualisation and image fusion • Freeware versions of Osirix MD, which is certified for clinical use
ImageJ [36]	• Extract mesh based on intensity isosurface using 3D viewer plug-in	• 2D image processing platform with 3D viewer plug-in

Seg3D has both manual and automatic segmentation tools and provides a library of add-ons with additional algorithms and applications optimised for particular segmentation applications. A key feature is that the interface makes it possible to visualise images in 3D with multiple volumes managed as layers. This facilitates the manipulation of several segmentations, which is particularly useful when it is necessary to use a combination of segmentation techniques to obtain the final surface. For example, the images may be cropped prior to more advanced segmentation processes in order to isolate the volume of interest. Furthermore, it provides Boolean transforms to combine multiple segmentations into a single surface. Seg3D also provides the option of exporting the final segmentation to the STL file format.

3D Slicer has a multitude of other image manipulation options and can be used to register different scans to each other. Because the large range of tools provided by the software, the interface is more difficult to master. However, it provides a range of powerful segmentation algorithms and has a unique selection of extensions available, which can be utilised for more specific tasks.

The segmentations formed the basis of the 3D printed models, which we created using FDM, allowing easy, low-cost creation of anatomical models. It was possible to create the segmented structures with high detail, allowing them to be used as teaching models. Furthermore, the printed model of the ribs was found to be functionally close to a real rib cage when imaged by an ultrasound scanner [31].

Using FDM for the creation of anatomical models has limitations inherent to the printing technique: The surface of the models was rough and rigid, which is not a realistic representation of the real tissue, and support material has to be carefully removed without damaging the finished print. However, there is flexible PLA available and the model surface can be smoothed using 3D print coatings (e.g. XTC-3D by Smooth-On, Macungie, USA). An alternative approach is to use a material jetting technique, which can combine different polymers seamlessly in one print, offering the possibility of creating a gradient of flexibility.

## Conclusions and future work

We have introduced a general workflow that can be used to generate 3D printed anatomical models from medical imaging data. This streamlined pipeline is applicable for volumetric medical imaging data and works for a wide variety of organs and other anatomical regions of interest. We have demonstrated its use in the creation of models of ribs, a liver and a lung from CT datasets.

Recent developments in image segmentation algorithms have enabled the use of a multitude of tools and strategies in delineating anatomical structures of interest. We have provided an overview of the most relevant open-source tools that can be used for anatomical structure segmentation by end-users who are not medical or image processing specialists.

Future work will focus on creating flexible phantoms and exploring different materials with regard to their tissue mimicking characteristics in US and MRI systems.
